# Maternal–Fetal Exposome and Early-Life Microbiota–Pattern Recognition Receptors Signaling in Inflammatory Bowel Disease Susceptibility: A Mechanistic Review

**DOI:** 10.3390/ijms27198458

**Published:** 2026-09-23

**Authors:** Krzysztof Jurkiewicz, Sylwia Smolinska-Wilczynska

**Affiliations:** Department of Clinical Immunology, Faculty of Medicine, Wroclaw Medical University, Parkowa 34, 51-616 Wroclaw, Poland; krzysztof.jurkiewicz@student.umw.edu.pl

**Keywords:** inflammatory bowel disease, early-life exposome, maternal microbiota, infant microbiome, pattern-recognition receptors, Toll-like receptors, histamine, H2 receptor, trained immunity

## Abstract

Inflammatory bowel disease (IBD) develops through interactions among genetic susceptibility, intestinal microbiota, environmental exposures, and dysregulated mucosal immunity. Increasing evidence suggests that these interactions may begin before diagnosis—and possibly before birth—when maternal signals, microbial assembly, epithelial maturation, and immune education intersect. This narrative review synthesizes findings from human observational and cohort studies, experimental animal and cellular models, and mechanistic literature to examine how prenatal and early-life exposures may influence later IBD susceptibility. Human cohort studies associate later IBD with several early-life exposures, particularly repeated antibiotic use, whereas evidence for dietary, delivery, and household factors remains heterogeneous and vulnerable to confounding. Studies of pregnancies affected by IBD identify altered maternal and infant microbial and immune features but do not establish causality. Experimental models provide stronger mechanistic evidence that maternal microbial signals influence offspring immune development, neonatal epithelial cells acquire context-dependent tolerance, and microbial metabolites regulate regulatory T cells, type 17 immunity, barrier function, and chromatin-modifying enzymes. Histamine-receptor signaling further illustrates receptor-specific modulation of bacterial TLR (Toll-like receptors) responses in established IBD, although its role in early-life programming remains unproven. We propose a testable “developmental inflammatory set-point” model in which cell type, tissue compartment, timing, signal intensity, and host genotype determine whether microbial sensing promotes tolerance or persistent inflammatory readiness. This model is biologically plausible but remains unvalidated as a predictor of human IBD.

## 1. Introduction

Inflammatory bowel disease (IBD), comprising Crohn’s disease (CD) and ulcerative colitis (UC), is a multifactorial disorder resulting from dysregulated interactions between host susceptibility, the intestinal microbiota, and environmental factors. Although more than 200 susceptibility loci have been identified, genetic predisposition alone does not explain disease development. Increasing evidence indicates that impaired epithelial barrier function, altered microbial sensing, dysregulated immune responses, and disturbances in intestinal microbial communities act together in determining IBD susceptibility [1,2].

Early life represents a critical period in which the intestinal microbiota, epithelial barrier, and mucosal immune system undergo coordinated maturation. Prenatal, perinatal, and postnatal factors—including maternal health, delivery mode, feeding, antibiotic exposure, infections, and diet—can influence this developmental process and have been associated with subsequent IBD risk [3,4]. However, the reported associations are heterogeneous, and the mechanisms by which transient early-life exposures may produce persistent effects on intestinal immune function remain incompletely understood.

Here, we propose the concept of a developmental inflammatory set point, defined operationally as the context-dependent threshold and magnitude of intestinal tissue responses to subsequent microbial or inflammatory stimuli, shaped during early life by interactions among microbial succession, epithelial maturation, PRR signaling, metabolic cues, immune-cell differentiation, tissue repair, and host genotype [3,5,6,7,8]. The set point is therefore conceived as a dynamic and potentially reversible functional state, rather than a fixed developmental imprint, biomarker, or established determinant of IBD [3,5,6,7,8]. Importantly, the persistence of such developmental effects may also involve epigenetic regulation, including DNA methylation, histone modifications, and regulatory RNAs such as microRNAs (miRNAs), which can modify gene expression and cellular responsiveness without altering the underlying DNA sequence. These mechanisms provide a potential molecular basis by which transient early-life environmental or microbial signals could produce longer-lasting changes in epithelial and immune-cell states. In this review, we examine how maternal–fetal signals, microbial succession, PRR signaling, microbial metabolites, epigenetic mechanisms, and immunometabolic adaptation may contribute to this process, while distinguishing established experimental mechanisms from human associations and conceptual inference. The proposed framework is intended to identify potential mechanistic links between early-life exposures and later IBD susceptibility rather than to represent an established biomarker or physiological variable [5,6,9,10,11,12,13]. The early-life factors and mechanisms potentially linking developmental processes with later IBD susceptibility are summarized in Table 1.

## 2. Literature Search Strategy and Evidence Framework

This work is a narrative review supported by a structured literature search rather than a systematic review or meta-analysis. PubMed/MEDLINE was searched from database inception to 25 August 2026, supplemented by backward citation screening and targeted searches of publisher databases. Search concepts combined terms for IBD, Crohn’s disease, ulcerative colitis, pregnancy, maternal exposure, infancy, childhood, microbiota or microbiome, antibiotics, diet, delivery mode, breastfeeding, TLR, Nucleotide-binding Oligomerization Domain (NOD), inflammasome, PRR, short-chain fatty acid (SCFA), bile acid, tryptophan, histamine, histamine receptor, biogenic amine, epigenetic programming, immune tolerance, and trained immunity. Peer-reviewed human studies, mechanistic animal or organoid studies, and authoritative reviews in English were considered. Priority was given to prospective human studies and publications from 2020 to 2026, while older experiments were retained when they established a mechanism that remains foundational.

The evidence is interpreted on four levels. “Direct human longitudinal evidence” denotes measurement of an exposure before a later IBD diagnosis. “Human mechanistic evidence” denotes molecular, microbial, or immune measurements in mothers, cord blood, infants, or children without proof of subsequent IBD. “Experimental causal evidence” denotes a controlled perturbation in animals, organoids, or cell systems. “Conceptual inference” denotes a biologically reasoned connection that has not been tested as a complete pathway. These levels are deliberately not collapsed into a single strong/moderate/weak score: strong evidence that butyrate promotes regulatory T-cell differentiation, for example, is not equivalent to evidence that low neonatal butyrate causes adult IBD.

This evidence framework was selected specifically to prevent mechanistic findings from experimental systems from being interpreted as direct evidence of developmental programming of human IBD. It also allows the present review to identify the specific evidentiary gaps that must be bridged before the proposed developmental inflammatory set point can be considered a measurable determinant of disease susceptibility.

## 3. Developmental Windows and Human Evidence

### 3.1. Prenatal and Early-Life Exposures Associated with IBD

The prenatal period, birth, infancy, and early childhood form overlapping rather than sharply separated developmental windows. During these periods, intestinal microbial succession, epithelial maturation, immune-cell development, and metabolic adaptation occur in parallel. The consequences of an exposure therefore depend on its timing relative to these processes, as well as on subsequent microbial recovery and host context [1,5,6,7,14,15,16,17].

Among candidate factors, childhood antibiotic exposure has one of the more reproducible epidemiological associations with IBD. A large Danish nationwide cohort found that antibiotic exposure was associated with incident IBD across age groups, with the highest rates after repeated courses and after agents commonly used for gastrointestinal pathogens [18]. In pooled Scandinavian birth cohorts, early infections and antibiotic use were both examined; antibiotic exposure remained associated with later childhood or early-adult IBD after accounting for infection frequency, although residual confounding cannot be excluded [19]. A systematic review and meta-analysis of pediatric IBD risk factors similarly identified antibiotic exposure, passive smoking, higher socioeconomic status, and high intake of sweetened foods as relevant signals, while the certainty and consistency differed by factor [4].

Antibiotic associations should not be interpreted as a simple drug-to-disease causal chain. Antibiotics may mark infection burden, altered immune development, genetic susceptibility, or early symptoms of undiagnosed disease. Spectrum, cumulative dose, age at exposure, and ecological recovery are rarely captured with equal precision. Reverse causation is especially plausible near diagnosis. Studies that impose lag periods, compare indications, analyze dose–response patterns, and measure microbial recovery are therefore more informative than a binary “ever exposed” variable [18].

Maternal diet is biologically attractive because it can alter maternal metabolism, inflammation, and microbial products without requiring direct fetal colonization. In the Norwegian Mother, Father and Child Cohort Study, higher dietary diversity during pregnancy was associated with a lower risk of UC in offspring, but not clearly with overall IBD or CD; the number of affected offspring was limited and the finding requires replication [20]. This result supports further study of dietary patterns rather than individual nutrients, but it does not justify a preventive prescription. Similarly, smoking exposure, urbanization, household composition, pets, sanitation, and childhood diet may alter immune or microbial development, yet their associations vary across populations and may reflect correlated social and behavioral factors [3,4].

Delivery mode and breastfeeding have strong effects on the early microbiota, but evidence linking them directly to IBD is less consistent than evidence linking them to microbial composition. Caesarean birth often delays acquisition of maternal gut-associated strains and increases early skin- or hospital-associated taxa, while breastfeeding favors HMO-utilizing bifidobacteria. Many compositional differences attenuate with age, and neither exposure is deterministic [6,17]. Clinical decisions about delivery or feeding should therefore not be reframed around unproven IBD prevention claims.

### 3.2. Very-Early-Onset and Paediatric IBD as a Developmental Phenotype

Very-early-onset IBD (VEO-IBD), generally defined as IBD diagnosed before 6 years of age, provides a clinically informative phenotype in which developmental timing, host genetic susceptibility, and early-life environmental exposures converge within a narrow biological window. Compared with later-onset IBD, VEO-IBD is enriched for monogenic forms involving defects in epithelial integrity, innate microbial sensing, bacterial clearance, adaptive immunity, and immune regulation, although most children with VEO-IBD do not have an identifiable monogenic cause [21]. Thus, VEO-IBD should not be regarded as synonymous with monogenic IBD; rather, it represents a heterogeneous phenotype in which Mendelian susceptibility, common polygenic risk, and developmental environmental influences may coexist.

This distinction is directly relevant to the developmental inflammatory set-point framework proposed here. In children with VEO-IBD, the interval between developmental immune maturation and clinical disease onset is relatively short, potentially reducing the temporal gap between early-life exposures, establishment of host–microbiota interactions, and manifestation of intestinal inflammation. In monogenic forms, the underlying defect may alter how microbial signals are sensed or contained from the earliest stages of life, whereas in polygenic VEO-IBD, multiple susceptibility variants may modify the response to microbial succession, antibiotics, infections, diet, or maternal signals without determining disease on their own. The same early-life exposure may therefore have different consequences depending on developmental stage and host genotype, providing a clinically relevant setting in which to examine gene–exposome interactions and developmental calibration of mucosal responsiveness.

Importantly, the existence of VEO-IBD does not establish that early-life exposures cause disease. Rather, this phenotype offers a high-information context for testing the proposed model: prospective studies beginning before disease onset could determine whether early-life exposures are associated with persistent changes in microbiota, metabolites, PRR responsiveness, epithelial function, or immune-cell states, and whether these changes differ according to monogenic or polygenic susceptibility. Such studies could help distinguish a developmental modifier of inflammatory responsiveness from a primary genetic defect or from transient preclinical inflammation.

### 3.3. Offspring of Mothers with IBD as a High-Information Model

Pregnancy in women with IBD combines genetic susceptibility with variation in inflammation, diet, medication, delivery mode, and maternal microbiota. It is consequently a valuable—but analytically complex—setting in which to study developmental mechanisms. Torres and colleagues reported that infants born to mothers with IBD had altered microbial profiles; transfer of maternal or infant microbiota to germ-free mice produced adaptive immune abnormalities, providing experimental evidence that the microbial communities could influence immunity [22]. The study did not show that the infants later developed IBD, and the mouse transfer model cannot isolate genetics, maternal disease activity, medication, and postnatal environment.

A prospective human cohort identified differences in infant microbiota and breast-milk composition in the context of maternal IBD [23], while a focused review has synthesized how maternal inflammation and immunomodulator exposure may affect infant immune development [16]. These observations strengthen the case for a maternal–infant biological interface but also demonstrate why “maternal IBD” cannot be treated as a uniform exposure. Active inflammation may alter cytokines and nutrient handling; immunomodulators or biologics can cross the placenta to varying degrees; feeding and delivery practices may differ; and familial risk reflects inherited and shared environmental components. Appropriate analyses require repeated measures of disease activity, drug exposure, maternal diet, microbiota, metabolome, and infant development rather than a single case–control comparison.

### 3.4. From Association to Causality

Three causal gaps remain. First, most birth cohorts are too small or too short to capture enough IBD diagnoses, a relatively uncommon outcome that may occur decades later. Second, early microbial measurements are highly dynamic; a difference at one month may represent delayed maturation rather than a stable pathological state. Third, a later diagnosis may be preceded by years of subclinical changes. Prospective studies of established IBD risk populations show that microbial, immune, and metabolic alterations can occur before clinical disease [24,25], but these preclinical signatures should not be assumed to originate in infancy.

Mendelian randomization (MR) can test whether genetically predicted microbial traits are associated with IBD, but current microbial genome-wide association studies have limited taxonomic resolution and substantial population heterogeneity. MR estimates generally represent lifelong genetic predisposition to a microbial feature, not exposure during a defined developmental window [26]. Triangulation is therefore necessary: epidemiology can establish temporality, experimental models can test mechanism, and serial human molecular phenotyping can determine whether the relevant state persists and mediates disease risk.

## 4. Maternal Microbiota, the Placental Interface, and Transmitted Signals

### 4.1. Colonization Is Not Required for Prenatal Microbial Influence

Claims of a stable resident microbiome in the healthy placenta or fetus remain controversial because low-biomass samples are highly vulnerable to reagent and environmental contamination. Current evidence does not support routine colonization of the healthy fetus by a complex viable microbiota [27]. This conclusion does not imply immunological isolation. Prenatal microbial influence can occur without fetal colonization through maternal microbial exposure and the transfer of microbial-derived products or signals. Maternal microbial molecules, metabolites, microbial antigens, antibodies, cytokines, and nutrients can reach or modify the placental–fetal interface, while maternal inflammation can alter placental function. The scientifically defensible model is therefore prenatal exposure to maternal microbial signals and, where demonstrated, transplacental transfer of microbial products, followed by extensive acquisition of viable microorganisms at and after birth, rather than a presumed fetal gut microbiome.

In a landmark mouse study, transient gestational colonization of germ-free dams altered innate immune development in offspring. Maternal microbiota-derived molecules reached the offspring and influenced intestinal immune gene expression and innate-cell populations [9]. This study demonstrates the biological potential of maternal microbial signaling without requiring stable prenatal fetal colonization, but dose, route, microbial composition, and placental physiology differ from human pregnancy. Reviews of human and animal data similarly conclude that maternal gut, vaginal, oral, and milk-associated communities may influence offspring through multiple routes, including microbial products, metabolites, immune mediators, and direct postnatal transmission of viable microorganisms, with the strongest direct evidence supporting postnatal microbial transmission and metabolite- or immune-mediated effects [14,15].

### 4.2. Candidate Molecular Mediators

SCFAs, indole derivatives, bile acids, and other small molecules are candidate mediators because they connect microbial metabolism with host receptors and chromatin-modifying enzymes. These compounds should be distinguished from viable microbial transfer: their biological effect would reflect exposure to microbial-derived products or metabolites rather than prenatal colonization by living organisms. Maternal concentrations, placental transport, fetal metabolism, and tissue exposure, however, are not interchangeable. Demonstration that a metabolite is present in maternal blood or that it affects fetal cells in vitro does not establish physiologically relevant transplacental signaling. The most informative studies combine quantitative metabolomics in maternal and cord blood with placental transport measurements and a perturbation that changes offspring immune development.

Maternal antibodies provide another route. IgG is actively transported across the placenta and can bind microbial antigens, while secretory IgA in milk shapes postnatal microbial containment [15]. These mechanisms may buffer neonatal exposure, promote antigen sampling, or, in disease-specific contexts, transmit altered immune recognition. Such effects are likely antigen- and Fc-dependent and should not be generalized as uniformly protective or harmful. Maternal cytokines and acute-phase signals may also alter fetal hematopoiesis or placental barrier functions, but separating direct fetal programming from preterm birth, altered growth, or postnatal care remains difficult [16].

### 4.3. Maternal–Infant Microbial Continuity After Birth

Evidence for postnatal transmission of viable maternal microorganisms is substantially stronger than evidence for prenatal fetal colonization. Delivery exposes the infant to maternal intestinal, vaginal, skin, oral, and environmental microorganisms, providing an important source of viable microbial strains during the initial establishment of the gut ecosystem. Breastfeeding subsequently provides both microorganisms and selective substrates, particularly human milk oligosaccharides (HMOs), that favor taxa adapted to the infant gut. Longitudinal analyses show that microbiota maturation follows recognizable trajectories while retaining substantial inter-individual variation; delivery-associated differences are often greatest early in life and subsequently attenuate [17]. This trajectory-based view is more informative than treating individual taxa as universally beneficial or pathogenic.

Maternal disease may perturb microbial transmission by altering maternal source communities, delivery practices, antibiotic exposure, diet, or milk composition. However, the presence of an altered maternal source community does not predict successful transmission or stable infant colonization. Strain fitness, bacteriophages, oxygen tension, feeding, host glycans, immune pressure, and competition with established microorganisms all influence whether transmitted strains successfully engraft and persist. Accordingly, studies should distinguish maternal microbial exposure from successful transmission and persistent colonization and should resolve strains and functions rather than relying only on 16S-defined genera. Observed differences should also be interpreted in terms of whether they reflect delayed microbiome maturation, altered microbial functions, or acquisition of pathobiont traits.

Importantly, maternal–infant microbial continuity should not be interpreted simply as transmission of fixed bacterial identities with predetermined biological effects. Strain-level genomic plasticity can alter microbial phenotypes through gene acquisition or loss, mobile genetic elements, bacteriophage interactions, and other mechanisms of functional adaptation, allowing closely related strains to differ substantially in metabolic capacity, host interaction, and inflammatory potential [18]. Consequently, the same microbial lineage may occupy different functional states along a spectrum from commensalism and mutualism to pathobiont or pathogenic behavior, depending on host genotype, nutrient availability, immune pressure, ecological competition, and epithelial conditions. This context dependence is particularly relevant during early life, when the intestinal ecosystem is rapidly changing. Maternal transmission may therefore provide not only microbial taxa but also strains with distinct genomic and functional potential, while the infant environment can subsequently select for phenotypes that promote persistence, immune tolerance, or, under specific conditions, inflammatory activity. Strain-resolved metagenomics and longitudinal functional profiling may therefore be required to determine whether maternal–infant continuity represents transfer of stable beneficial functions, transmission of latent pathogenic potential, or dynamic adaptation during microbiome maturation [18].

## 5. Co-Development of Infant Microbiota, Barrier, and Mucosal Tolerance

### 5.1. Ecological Succession and Epithelial Maturation

The newborn intestine changes rapidly in oxygen availability, nutrient supply, mucus structure, antimicrobial peptides, epithelial turnover, and immune-cell composition. Early facultative anaerobes consume oxygen and help create conditions for obligate anaerobes. Milk feeding selects organisms capable of using HMOs and host-derived glycans; complementary foods expand carbohydrate fermentation and microbial metabolic diversity. By early childhood, community structure becomes more adult-like, although maturation continues and varies among individuals [17,28].

The epithelial barrier is not merely a passive wall. Intestinal epithelial cells (IECs) integrate microbial, dietary, neural, and immune signals; secrete mucus, antimicrobial peptides, and cytokines; transport IgA; and coordinate repair. Specialized Paneth, goblet, enteroendocrine, tuft, and microfold cells perform distinct sensing and effector functions. Tight-junction proteins regulate paracellular permeability, but barrier integrity also depends on mucus organization, cell turnover, antimicrobial spatial segregation, and immune containment. A transient increase in permeability is therefore not synonymous with a durable “leaky gut,” and composition-only microbiome data cannot establish barrier failure [10].

These developmental changes are relevant to the proposed inflammatory set point because epithelial maturation and microbial succession occur simultaneously and are therefore likely to establish the initial conditions under which later microbial signals are interpreted. A perturbation during this period may consequently have different consequences from an equivalent perturbation in an immunologically mature intestine. The relevant outcome is not necessarily persistent dysbiosis, but a change in the trajectory of host–microbe adaptation, including the threshold for epithelial activation, tolerance, repair, and inflammatory recruitment [5,7,8,17].

### 5.2. Development of Tolerance

Early microbial encounters expand regulatory networks that distinguish colonization from invasion. Regulatory T (Treg) cells, tolerogenic antigen-presenting cells, IgA-producing B cells, and tissue-resident innate populations develop in response to microbial antigens and metabolites. In mice, RORγt-expressing (retinoic acid receptor-related orphan receptor gamma t) antigen-presenting cells instruct microbiota-specific peripheral Treg differentiation during a defined early-life window [29]. This work identifies a developmental checkpoint, but the precise human cell counterparts, timing, and disease consequences remain under investigation.

Bifidobacteria provide an example of a microbial function rather than a universal taxonomic marker. In breastfed infants, efficient HMO utilization can generate acetate and lactate, lower luminal pH, restrict potential pathogens, and supply cross-feeding substrates. A human infant study linked bifidobacteria and their metabolites with immune imprinting and reduced markers of intestinal inflammation [30]. Nevertheless, effects are strain- and substrate-dependent, and abundance alone does not capture metabolic output.

Treg and type 17 programs should not be treated as a binary switch. RORγt+ Treg cells can support tolerance to commensals, while homeostatic Th17 cells contribute to barrier defense. Pathogenic Th17-like states emerge under specific cytokine and tissue contexts and can show plasticity toward interferon-γ-producing phenotypes [31]. Thus, a higher frequency of IL-17-producing cells is not by itself proof of harmful programming; location, antigen specificity, transcriptional state, and concurrent regulatory function are decisive.

### 5.3. Resilience After Perturbation

An ecologically meaningful early-life perturbation has at least three dimensions: immediate displacement, recovery trajectory, and functional compensation. Antibiotics may reduce susceptible taxa, alter resistance genes and metabolite production, and open ecological niches. Some communities recover in composition but not function, whereas others converge toward age-expected states. Repeated perturbations may be more consequential than a single exposure if they occur during diet transition or immune checkpoints. Conversely, developmental plasticity can permit recovery.

This resilience framework helps avoid deterministic language. Caesarean birth, formula feeding, infection, or antibiotic treatment do not encode a fixed outcome. Their potential relevance lies in changing the probability, order, or duration of microbial functions during a period of host plasticity. Future IBD studies should therefore use age-normalized maturation scores, strain-resolved metagenomics, metabolomics, and within-child trajectories, alongside clinical indications and recovery-promoting exposures [3,17].

## 6. Developmental PRR Signaling

### 6.1. Cell Type, Localization, and Signal History Determine Outcome

PRRs recognize conserved microbial structures, but their outputs are not intrinsically pro- or anti-inflammatory. TLRs at the cell surface or in endosomes signal through adaptor combinations including MYD88, TIRAP, TRIF, and TRAM. NOD1 and NOD2 are cytosolic sensors that signal primarily through receptor-interacting serine/threonine-protein kinase 2 (RIPK2). Inflammasome-forming sensors, including NLRP3, assemble caspase-1-activating complexes that process IL-1β and IL-18 and can induce gasdermin-dependent pyroptosis. These are related layers of innate surveillance, not interchangeable branches [32,33].

In the proposed framework, developmental PRR signaling represents one of the principal molecular mechanisms through which the inflammatory set point could be established. The relevant variable is unlikely to be constitutive receptor expression alone. Rather, the set point may emerge from the integrated relationship between receptor localization, signal intensity, prior exposure, negative-feedback pathways, epithelial differentiation, metabolic state, and the cellular composition of the responding tissue. Thus, two individuals with similar microbial exposure could acquire different response thresholds if developmental PRR calibration occurs in different genetic or metabolic contexts.

In the intestine, the same ligand can produce different results in an IEC, monocyte, dendritic cell, stromal cell, or lymphocyte. Apical sensing may reinforce containment, whereas basolateral access can indicate barrier breach. Low or repeated exposure can induce negative regulators and hyporesponsiveness; acute high-intensity exposure can activate inflammatory and cell-death pathways. PRR signaling also supports homeostasis by inducing antimicrobial peptides, epithelial proliferation, mucus production, and repair [10,34]. Any developmental model must therefore specify receptor, cell type, subcellular or tissue compartment, ligand, dose, and exposure history.

### 6.2. TLR2: Microbial Context and Heterodimer Specificity

TLR2 forms heterodimers with TLR1 or TLR6 and recognizes a range of bacterial lipoproteins and cell-wall components. In IECs and myeloid cells, TLR2 signaling can enhance barrier functions and antimicrobial defense, yet excessive or misplaced activation can amplify cytokine production. Ligand preparations differ in purity and co-activation of other receptors, complicating comparison across experiments. Experimental evidence indicates that TLR2 can influence immune maturation during early life, but the available developmental data are derived primarily from mouse models. In mouse pups, maternal TLR2 deficiency during lactation impaired the development of oral tolerance, with reduced tolerogenic dendritic cells and Treg cells, increased intestinal permeability, and altered milk cytokine and growth-factor composition. These findings provide experimental causal evidence that TLR2-dependent maternal–neonatal signaling can modify early immune development, but they do not establish that an analogous developmental program occurs in human neonates or that it determines subsequent IBD risk. Early-life studies rarely measure TLR2 signaling longitudinally in a cell-specific manner, so claims that a particular neonatal TLR2 state predicts IBD remain speculative [10,32,33].

The most plausible programming mechanisms are repeated ligand exposure, changes in receptor localization or negative regulators, and metabolite-dependent modification of downstream chromatin. These should be tested using purified ligands, defined microbial strains, intestinal organoid–immune co-cultures, and age-matched cells. Functional readouts—barrier repair, cytokine dynamics, antimicrobial production, and tolerance upon rechallenge—are more informative than receptor abundance alone [8,11,32,35].

### 6.3. TLR4 and Acquisition of Neonatal Endotoxin Tolerance

TLR4 recognizes lipopolysaccharide (LPS) in a complex with MD-2 and CD14. The pathway can signal from the plasma membrane through MYD88-dependent modules and from endosomes through TRIF-dependent modules. In an influential mouse study, fetal IECs responded strongly to LPS, whereas postnatal microbial exposure induced epithelial hyporesponsiveness associated with degradation and reduced expression of IRAK1 [7]. This provides direct experimental evidence for developmentally acquired epithelial endotoxin tolerance.

Several qualifications are essential. First, the mechanism was demonstrated in mice and should not be assumed to occur with identical timing in human neonates. Second, IEC tolerance does not imply systemic immunosuppression; myeloid cells may retain or acquire different response profiles. Third, LPS structure varies among bacterial species and can differ markedly in TLR4 agonism or antagonism. Finally, tolerance can be adaptive by preventing inflammation in a newly colonized intestine, whereas excessive suppression in a different compartment could impair pathogen defense. Developmental TLR4 programming is therefore a context-dependent calibration process, not a linear transition from “high inflammation” to “low inflammation” [5,7].

Within the proposed model, neonatal TLR4 tolerance therefore represents a candidate mechanism for shifting the inflammatory set point rather than simply suppressing inflammation. Whether such calibration is beneficial or maladaptive would depend on the subsequent microbial environment, the reversibility of the tolerant state, and the ability of other intestinal compartments to maintain effective pathogen defense [5,8].

### 6.4. TLR5 and Flagellin Sensing

TLR5 recognizes extracellular flagellin and is expressed by epithelial and immune-cell populations. In the healthy intestine, spatial organization limits access of flagellin to responsive compartments, while TLR5-dependent signals can shape antimicrobial defense, IgA responses, and microbial ecology [36]. Loss or dysregulation of TLR5 produces model-dependent phenotypes, emphasizing interactions among host genotype, microbial community, and facility conditions.

Developmental evidence is available from neonatal mouse models. Age-dependent epithelial TLR5 expression during the neonatal period regulates flagellated bacterial colonization through TLR5-dependent antimicrobial responses and can influence gut microbiota composition beyond weaning. This constitutes experimental causal evidence that neonatal TLR5 signaling can shape host–microbiota development, but it does not demonstrate that the same developmental mechanism operates in human infants or that it directly modifies later IBD susceptibility.

Anti-flagellin antibodies are observed in subsets of CD and can reflect adaptive exposure to bacterial flagellar antigens. They do not, however, demonstrate that neonatal TLR5 signaling was developmentally programmed. Direct support would require early-life TLR5 functional measurements that precede disease, or perturbation experiments showing a persistent, TLR5-dependent phenotype. Thus, current evidence supports TLR5 as a developmentally active microbial-sensing pathway in experimental models, whereas its contribution to developmental IBD susceptibility remains unproven [33].

### 6.5. TLR9: Compartmentalized Sensing of Microbial DNA

TLR9 senses unmethylated CpG-rich DNA in endosomal compartments. In IEC models, apical and basolateral TLR9 stimulation can produce different outputs; apical signaling has been associated with homeostatic responses, whereas access from the tissue side may accompany barrier disruption [37]. These observations illustrate a broader principle: localization and trafficking can be as important as receptor expression.

Developmental evidence for TLR9 signaling comes mainly from experimental studies of the neonatal intestine. In a murine model of neonatal intestinal inflammation, TLR9 expression and signaling were developmentally regulated and activation of TLR9 reduced the severity of experimental necrotizing enterocolitis, whereas TLR9 deficiency increased disease severity. These findings constitute experimental causal evidence for a functional role of TLR9 in the neonatal intestine, but the model concerns neonatal intestinal injury/NEC rather than IBD and therefore cannot by itself establish developmental programming of human IBD susceptibility.

The literature does not support a universal claim that TLR9 activation is protective or pathogenic in IBD. Microbial DNA composition, endosomal processing, cell lineage, and co-stimulation all affect outcome. Developmental studies should distinguish epithelial from plasmacytoid dendritic-cell and other hematopoietic responses, and should avoid inferring in vivo programming from bulk-tissue TLR9 expression.

### 6.6. NOD1/NOD2–RIPK2 and Inflammasomes Are Distinct Modules

NOD1 and NOD2 recognize peptidoglycan-derived motifs in the cytosol and recruit RIPK2 to activate NF-κB and mitogen-activated protein kinase pathways. NOD2 is strongly relevant to CD genetics and links bacterial sensing with antimicrobial defense, autophagy, and epithelial homeostasis. In intestinal stem cells, NOD2 signaling can promote cytoprotection through mitophagy under stress [38]. Experimental evidence also links NOD2 to the consequences of neonatal environmental perturbation. In mice, neonatal antibiotic exposure produced more persistent microbiota alterations and increased susceptibility to subsequent experimental colitis in Nod2-deficient animals, indicating that NOD2 can influence microbial resilience and later inflammatory responses after an early-life perturbation. This represents experimental causal evidence in a murine model, but it does not establish that NOD2-dependent developmental calibration occurs in human infants or that neonatal NOD2 signaling directly determines human Crohn’s disease risk. Disease-associated NOD2 variants may therefore affect microbial handling and repair in addition to cytokine output.

Inflammasomes operate differently. Sensors such as NLRP3 assemble multiprotein complexes that activate caspase-1, mature IL-1β and IL-18, and may induce pyroptosis. Depending on cell type and timing, inflammasome activity can support barrier repair and pathogen control or drive tissue injury. NOD2 can interact with autophagy and inflammatory networks, but it should not be described as simply another inflammasome sensor. Studies of early-life programming should measure these modules separately and account for a two-signal architecture in canonical inflammasome activation: priming of pro-cytokines and inflammasome components, followed by an activation signal [10,33].

Experimental evidence for a developmental role of inflammasome signaling is also derived mainly from neonatal animal models. In neonatal mice subjected to experimental necrotizing enterocolitis, pharmacological inhibition of the NLRP3 inflammasome reduced intestinal inflammation and tissue injury, indicating that NLRP3 activity can contribute to early-life intestinal inflammatory pathology. This constitutes experimental evidence for a functional role of NLRP3 during neonatal intestinal injury, but it should not be interpreted as direct evidence of developmental programming of human IBD. Whether transient inflammasome activation during human early-life development produces persistent changes in inflammatory responsiveness remains unknown.

## 7. The Metabolite–Epigenome–Immunometabolism Axis

### 7.1. Short-Chain Fatty Acids

Acetate, propionate, and butyrate are generated through microbial fermentation of dietary or host-derived carbohydrates. They signal through G-protein-coupled receptors, alter cellular metabolism, and—in a concentration- and cell-dependent manner—inhibit histone deacetylases (HDACs). Seminal mouse experiments showed that butyrate and other commensal metabolites promote colonic or peripheral Treg generation [39,40]. SCFAs can also fuel colonocytes, support epithelial hypoxia, influence myeloid-cell function, and alter cytokine production [12].

If PRRs provide one layer of developmental immune calibration, microbial metabolites may provide a second layer by linking ecological succession to cellular metabolism and gene regulation. In this sense, the developmental inflammatory set point is not determined by microbial recognition alone. It may also reflect the metabolic environment in which PRR signals are received, including the availability of substrates and metabolites capable of modifying cellular energetics, receptor signaling, chromatin state, and regulatory lymphocyte differentiation [11,12,35,40,41,42].

These mechanisms are often compressed into the statement “more SCFA is beneficial,” which is too simple. Luminal and tissue concentrations differ, individual SCFAs have distinct receptor affinities, and effects depend on oxygen, substrate use, inflammatory state, and cell lineage. Fecal SCFA concentrations reflect production, absorption, and transit and are not a direct measure of epithelial exposure. In early life, milk-derived substrates and microbial cross-feeding create a metabolic context that differs from the adult fiber-fermenting colon. Human studies should therefore combine dietary intake, microbial genes, absolute metabolite concentrations, isotope tracing where feasible, and host-response markers [11,12,28].

### 7.2. Tryptophan Metabolites and Aryl Hydrocarbon Receptor Signaling

Microbial metabolism converts tryptophan into indoles and related compounds that can activate the aryl hydrocarbon receptor (AhR). In experimental systems, microbiota-derived tryptophan metabolites promote IL-22-dependent mucosal defense and help balance reactivity at barrier sites [41]. AhR signaling also intersects with IEC differentiation, innate lymphoid cells, Treg cells, and type 17 responses. The outcome depends on ligand identity, exposure kinetics, cell type, and concurrent cytokines.

Reduced microbial capacity for producing particular AhR ligands has been reported in inflammatory conditions, but cross-sectional depletion after disease onset cannot establish developmental causality. Maternal diet, infant feeding, microbial succession, and host enzymes jointly determine the tryptophan metabolite pool. Longitudinal studies should identify specific ligands rather than relying on total tryptophan or a single downstream cytokine.

### 7.3. Bile Acids

Primary bile acids synthesized by the host are deconjugated and transformed by intestinal microbes into a diverse secondary bile-acid pool. Bile acids signal through receptors including farnesoid X receptor (FXR) and Takeda G-protein-coupled receptor 5 (TGR5), and selected derivatives directly influence RORγt-dependent Treg and Th17 differentiation. Experimental work has identified bile-acid metabolites that reciprocally regulate these cell states [42], while broader reviews describe effects on myeloid cells, epithelial barriers, and systemic metabolism [43].

The neonatal bile-acid pool differs from that of adults because liver function, diet, enterohepatic circulation, and microbial transformation pathways are still developing. A metabolite that is mechanistically active in adult mice or at pharmacological concentration should not automatically be assigned a programming role in human infancy. Quantitative developmental reference ranges and tissue-specific receptor readouts are needed.

### 7.4. Histamine as a Microbiota–PRR Modulator

Histamine provides an additional example of how microbially linked metabolites can modify PRR outputs rather than act as uniformly pro-inflammatory signals. Histamine may originate from host immune cells, dietary sources, and histidine-decarboxylase-positive intestinal bacteria and signals through four receptors, H1R–H4R [44].

In peripheral blood mononuclear cells from healthy individuals, histamine suppressed cytokine secretion induced by TLR2, TLR4, TLR5, and TLR9 stimulation through H2R. This regulatory effect was diminished in patients with Crohn’s disease or ulcerative colitis, who also showed a reduced proportion of circulating H2R-expressing monocytes. Histamine-receptor expression was altered in inflamed intestinal mucosa, whereas H2R deficiency or pharmacological antagonism aggravated experimental colitis and increased mucosal IFN-γ- and IL-17-producing T cells [45]. These findings position histamine-receptor signaling at the interface between microbial sensing and mucosal inflammation. However, the human observations were obtained after IBD onset, and the experimental models did not address prenatal or infant exposure. Histamine therefore provides mechanistic support for receptor-specific modulation of bacterial PRR responses, but its contribution to the developmental inflammatory set point remains a testable hypothesis.

### 7.5. Epigenetic and Immunometabolic Persistence

Epigenetic regulation—including DNA methylation, histone modification, chromatin accessibility, and regulatory RNA—can stabilize cell states without changing DNA sequence. Cellular metabolites influence these processes: acetyl-CoA supplies acetyl groups; S-adenosylmethionine supports methylation; α-ketoglutarate, succinate, and fumarate regulate dioxygenases; and NAD+ availability affects sirtuins. Microbial metabolites can therefore alter gene regulation directly or through host metabolic pathways [5,35].

“Trained immunity” refers to a persistent change in innate-cell responsiveness after an initial stimulus, mediated by metabolic and epigenetic remodeling. It is distinct from adaptive antigen-specific memory. Related states include tolerance, in which secondary responses are reduced, and priming, in which a transient first signal enhances a later response. The terminology should be reserved for experiments that include an initial stimulus, a resting interval, a secondary challenge, and evidence of persistence beyond immediate activation [8,46].

Persistence presents an additional biological problem because circulating monocytes are short-lived. Long-term trained states may be maintained through hematopoietic stem and progenitor cells or long-lived tissue macrophages. IECs can also retain inflammatory or regenerative memory through stem-cell chromatin, but this is not identical to myeloid trained immunity. In IBD, trained or tolerant states are an emerging framework rather than an established early-life cause [13]. Cell lineage, duration, reversibility, and functional consequences should be demonstrated rather than inferred from a static epigenetic difference.

## 8. Gene–Exposome Interactions Relevant to IBD

Host genotype may determine the range within which the developmental inflammatory set point can be established and subsequently modified. Genetic susceptibility should therefore be viewed not simply as a background risk factor, but as a determinant of how epithelial and immune compartments interpret microbial and metabolic signals during developmental windows. This provides a mechanistic basis for gene–exposome interactions: the same exposure may produce different developmental consequences depending on the host’s capacity for microbial sensing, autophagy, glycosylation, metabolic adaptation, and tissue repair.

Genetic susceptibility is likely to modify both exposure effects and the host response to microbial succession. NOD2 illustrates this principle: risk variants can change cytosolic bacterial sensing, antimicrobial programs, autophagy, and epithelial repair. ATG16L1 links microbial handling with autophagy and Paneth-cell biology; CARD9 connects fungal and bacterial sensing to myeloid signaling and microbial metabolism; IL23R modifies type 17 and regulatory immune circuits; and FUT2 affects epithelial glycosylation and the nutrient landscape available to mucosa-associated microbes [2,47].

These genes do not define a single pathway or guarantee disease. Their penetrance depends on other variants, microbial functions, infections, diet, smoking, and stochastic events. The same early exposure may therefore have different consequences across genotypes. Conversely, microbial functions may compensate for a host defect in one ecological context and amplify it in another. Analyses that test only main effects can miss such interactions, while small studies that screen many gene–environment combinations are prone to false-positive findings.

A rigorous developmental study should pre-specify plausible interactions and measure an intermediate phenotype. As a proposed test of this hypothesis, a developmental NOD2-by-antibiotic interaction could be investigated using strain-resolved microbial recovery, RIPK2-dependent functional responses, and epithelial antimicrobial or repair measures. Similarly, a proposed CARD9-focused developmental study could integrate longitudinal assessment of fungal communities with measurements of tryptophan-derived AhR ligands to test whether this interaction contributes to developmental immune calibration. A similarly proposed FUT2-focused analysis could quantify secretor status, mucosal glycans, and HMO-related microbial functions to determine whether genotype-dependent differences modify responses to early-life microbial exposures. Such experimental designs could move beyond statistical interaction toward a testable mechanistic chain, but these interactions should be regarded as proposed research hypotheses rather than established developmental mechanisms [1,47].

## 9. A Testable Developmental Inflammatory Set-Point Model

The evidence reviewed above supports a unifying hypothesis in which heterogeneous early-life exposures may alter the threshold and magnitude of later intestinal responses to microbial or inflammatory stimuli. The framework is therefore testable only if it generates observations that distinguish a persistent, developmentally conditioned change in response from transient exposure effects, age-related maturation, or nonspecific inflammation. The model proposes that these effects emerge from the interaction of developmental timing, microbial succession, epithelial and immune maturation, PRR calibration, metabolic signals, host genotype, and the capacity for ecological and tissue recovery [3,5,47]. Table 2 outlines testable predictions of the proposed model, candidate operational readouts, and observations that would weaken or falsify these predictions.

The position of this functional state may change over time because subsequent exposures can reinforce, compensate for, or overcome earlier developmental effects. Its position at any given time would reflect both the history of prior signals and the capacity of the developing host–microbiota ecosystem to recover from perturbation. Early-life antibiotics, maternal inflammatory signals, altered microbial transmission, dietary changes, or infection may influence this trajectory through different molecular routes, while subsequent exposures may either reinforce, compensate for, or overcome the resulting state [3,5,16,17].

Critically, the model does not imply that an altered developmental set point is equivalent to IBD or that early-life perturbation determines disease. Rather, it predicts that developmental calibration may modify the probability and magnitude of later responses to genetically and environmentally relevant challenges. IBD would therefore emerge when a susceptible host, an altered or insufficiently resilient developmental state, and subsequent inflammatory or microbial exposures converge over time [3,5,47]. The proposed developmental model, integrating maternal–fetal exposures, postnatal microbial succession, PRR signaling, and mucosal immune programming, is illustrated in Figure 1.

As illustrated in Figure 1, the proposed sequence can be represented as a developmental cascade: maternal and early-life exposures → altered microbial succession and host metabolic signals → cell- and compartment-specific PRR calibration → epithelial, innate, and adaptive immune adaptation → establishment or modification of the developmental inflammatory set point → altered resilience to subsequent perturbations → differential susceptibility to IBD. Each transition in this cascade represents a potential point at which genetic variation, exposure timing, signal intensity, or ecological recovery may modify the trajectory. Most early-life perturbations are expected to be transient or compensated; persistent susceptibility would require convergence of developmental timing, host vulnerability, incomplete recovery, and reinforcing later exposures [3,5]. Table 3 summarizes representative evidence supporting individual components of the proposed developmental pathway, together with the evidence types and principal limitations.

The evidence matrix in Table 3 shows why the complete model remains unproven. Direct human longitudinal evidence is strong for selected exposures such as repeated antibiotic use, while causal mechanistic evidence is strongest for individual receptor and metabolite pathways in experimental systems. The bridge between these evidence types—persistent, cell-resolved molecular mediation from infancy to later IBD—is largely missing.

## 10. Translational Implications and Research Priorities

### 10.1. What Should Not Yet Enter Clinical Practice

Current evidence does not justify altering necessary maternal IBD therapy, indicated antibiotics, mode of delivery, or infant feeding solely to prevent IBD. Active maternal disease itself carries risks, and medication decisions require individualized obstetric and gastroenterological assessment. Nevertheless, clinically relevant information about fetal drug exposure is increasingly available. Fetal exposure varies substantially between drug classes and depends on molecular properties, placental transport, gestational age, and treatment timing. Monoclonal antibodies containing an Fc region undergo increasing active transplacental transfer after approximately 14 weeks of gestation, with the majority of transfer occurring during the third trimester, whereas agents with limited placental transport, such as certolizumab pegol, result in substantially lower fetal drug concentrations [48,49]. Available cohort data are broadly reassuring for several commonly used IBD therapies, with no consistent signal of adverse pregnancy or early-childhood outcomes attributable to in utero exposure to anti-TNF agents, thiopurines, vedolizumab, or ustekinumab; however, evidence quality and duration of follow-up vary by drug [50,51]. The timing of the final antenatal biologic dose should therefore balance maintenance of maternal disease control against the anticipated degree of fetal drug transfer rather than follow a universal rule. In selected patients receiving anti-TNF therapy, administration of the final dose earlier in pregnancy may reduce neonatal drug concentrations, whereas continuation later in pregnancy may be appropriate when required to maintain remission [48,49].

Infant vaccination decisions should also be based on the specific agent and timing of in utero exposure. Inactivated vaccines should be administered according to routine schedules. For infants exposed in utero to biologics, recommendations concerning live vaccines have become more drug-specific: current international consensus supports consideration of live rotavirus vaccination in appropriate circumstances, whereas BCG should be avoided during the first 6 months after in utero anti-TNF exposure because of the reported risk of disseminated infection; where clinically necessary, decisions may incorporate the specific biologic, regional infection risk, and, in selected settings, measurement of infant drug concentrations [49]. These recommendations illustrate that prenatal IBD pharmacotherapy should be considered as a component of the developmental exposome, but they do not establish that in utero drug exposure itself causes later IBD.

At the same time, paediatric Crohn’s disease provides a clinical proof of principle that dietary and microbial manipulation can modify intestinal inflammation. Exclusive enteral nutrition (EEN), typically administered for 6–8 weeks, is recommended as a first-line induction treatment for active luminal Crohn’s disease in children and can promote clinical remission and mucosal healing [52]. Although the mechanisms are incompletely resolved, EEN can modify substrate availability, microbial composition and function, epithelial interactions, and metabolite production. EEN therefore provides an established clinical example that altering the diet–microbiota–host interface can influence mucosal inflammation, while also illustrating the distinction between an effective therapeutic intervention and the still-unproven hypothesis that early-life dietary or microbial exposures program future IBD susceptibility.

Commercial microbiome tests cannot currently identify a neonatal “IBD microbiome,” and empiric probiotics or metabolite supplementation cannot be assumed to reproduce strain-specific ecological functions. Prevention claims should therefore be held to a high standard because interventions during pregnancy and infancy affect both mother and child [3].

### 10.2. Prospective Cohort Design

The ideal study begins before or during pregnancy and follows offspring through the age of IBD risk. Maternal sampling should include disease activity, medication, diet, stool metagenomics, metabolomics, and blood inflammatory measures. Infant sampling should be dense around birth, feeding transitions, infections, and antibiotic courses, then repeated through childhood. Stool should be paired with host samples—blood, minimally invasive epithelial material, or validated mucosal surrogates—because microbiota data alone cannot define immune programming [3,22].

Analyses should use absolute microbial abundance, strain resolution, functional genes, metabolite quantification, and age-normalized trajectories. Clinical metadata must capture indication and timing of antibiotics, feeding, delivery, gestational age, household exposures, growth, and family history. Genotyping should be incorporated from the outset. Biobanking viable cells, microbial isolates, stool, plasma, and milk would permit later mechanistic testing as technology evolves [1,3,17,47].

### 10.3. Mechanistic Human Models

Human intestinal organoids derived from fetal, pediatric, and adult tissue can test age-dependent epithelial responses, but organoids lack the full microbial, immune, neural, and vascular environment. Co-culture with defined immune cells and microbes, microfluidic barrier systems, and carefully annotated pediatric tissues can improve physiological relevance. Experiments should compare apical and basolateral stimulation, repeated versus acute exposure, and recovery after withdrawal. CRISPR editing of NOD2, ATG16L1, CARD9, IL23R-related pathways, or FUT2 can test genotype-specific responses [1,33,47].

For epigenetic persistence, studies should include a washout or resting phase followed by heterologous and homologous rechallenge. Single-cell RNA sequencing alone is insufficient; chromatin accessibility, histone marks, DNA methylation where relevant, metabolic flux, and functional output should be integrated. Lineage tracing in animals and clonal or barcoding approaches in organoids can determine whether a memory state resides in stem/progenitor cells or differentiated populations [8,35,46].

### 10.4. Intervention Principles

An intervention should target a defined mechanism rather than a vague concept of dysbiosis. Examples include restoring a missing substrate–strain–metabolite function, preventing unnecessary ecological disruption, or correcting a receptor-specific barrier defect. Safety, developmental timing, engraftment, dose, and ecological context must be established. Outcomes should include both benefit and unintended impairment of pathogen defense or immune maturation [3,11,12,47].

Randomized prevention trials with IBD incidence as the endpoint will be difficult because of sample size and latency. A staged approach is more realistic: demonstrate target engagement and normalization of a validated intermediate; establish persistence and safety; then, test clinical outcomes in genetically or familially enriched populations. Adaptive designs and nested mechanistic trials within birth cohorts may accelerate this process [3].

## 11. Conclusions

The central implication of this review is that early-life exposures should be interpreted as potential modifiers of later mucosal responsiveness rather than as deterministic causes of IBD. Human evidence currently supports temporal associations for selected exposures, whereas mechanistic and causal support comes predominantly from experimental systems. The proposed developmental inflammatory set point provides a testable way to connect these evidence domains, but whether a persistent, measurable alteration in response thresholds mediates later human IBD risk remains unresolved.

Experimental studies provide causal support for individual components of the proposed pathway. Maternal microbial signals can influence offspring immune development; neonatal epithelial responses to LPS can acquire tolerance; NOD2–RIPK2 signaling connects bacterial sensing with autophagy and repair; and SCFAs, tryptophan derivatives, and bile-acid metabolites regulate barrier and lymphocyte programs through receptor, metabolic, and epigenetic mechanisms. Histamine–H2R signaling provides an additional example of receptor-specific modulation of TLR responses in established IBD, but developmental programming of this axis has not been demonstrated [44,45]. The direction of these effects depends on cell type, compartment, timing, dose, and prior signal history. NOD1/NOD2 signaling and inflammasome activation must be analyzed as distinct modules, and homeostatic type 17 responses must be distinguished from pathogenic states.

The developmental inflammatory set point proposed here provides a unifying, testable framework for understanding how heterogeneous early-life exposures may converge on later mucosal responsiveness. Rather than representing a single pathogenic pathway, it integrates developmental microbial succession, epithelial maturation, PRR calibration, metabolite-dependent signaling, immune-cell differentiation, host genotype, and the capacity for ecological and tissue recovery. Its central prediction is that early-life perturbations modify the threshold and magnitude of later intestinal responses to microbial or inflammatory stimuli, thereby altering susceptibility to IBD without directly determining disease. The key unresolved question is whether persistent, cell- and compartment-specific alteration in response thresholds can be demonstrated prospectively in humans and shown to mediate the relationship between early-life exposures and subsequent IBD. Serial multi-omics, genotype-aware causal analyses, functional challenge assays, and mechanism-specific interventions will be required to determine whether the developmental inflammatory set point represents a useful biological construct or ultimately a measurable determinant of disease susceptibility.

From a clinical perspective, the immediate value of this model is not to justify routine microbiome testing or preventive treatment in asymptomatic children, but to provide a framework for identifying potentially modifiable developmental exposures and defining individuals who may warrant closer longitudinal observation. If the proposed mechanisms are validated, clinical implementation could initially involve risk-stratified monitoring based on combinations of early-life exposure history, familial/genetic susceptibility, and validated microbiome or host-response biomarkers, rather than any single microbial signature. Such stratification could support earlier recognition of persistent gastrointestinal symptoms and facilitate prospective evaluation of targeted, mechanism-based interventions. However, antibiotic restriction, dietary modification, probiotic or microbial therapies, and other preventive strategies should not be recommended solely on the basis of the developmental inflammatory set-point hypothesis until their effects on long-term IBD risk are demonstrated in appropriately designed clinical studies. Thus, the practical contribution of the model is primarily to guide biomarker development, prospective risk stratification, and intervention trials aimed at preventing maladaptive inflammatory trajectories before clinically established IBD develops.

## Figures and Tables

**Figure 1:**
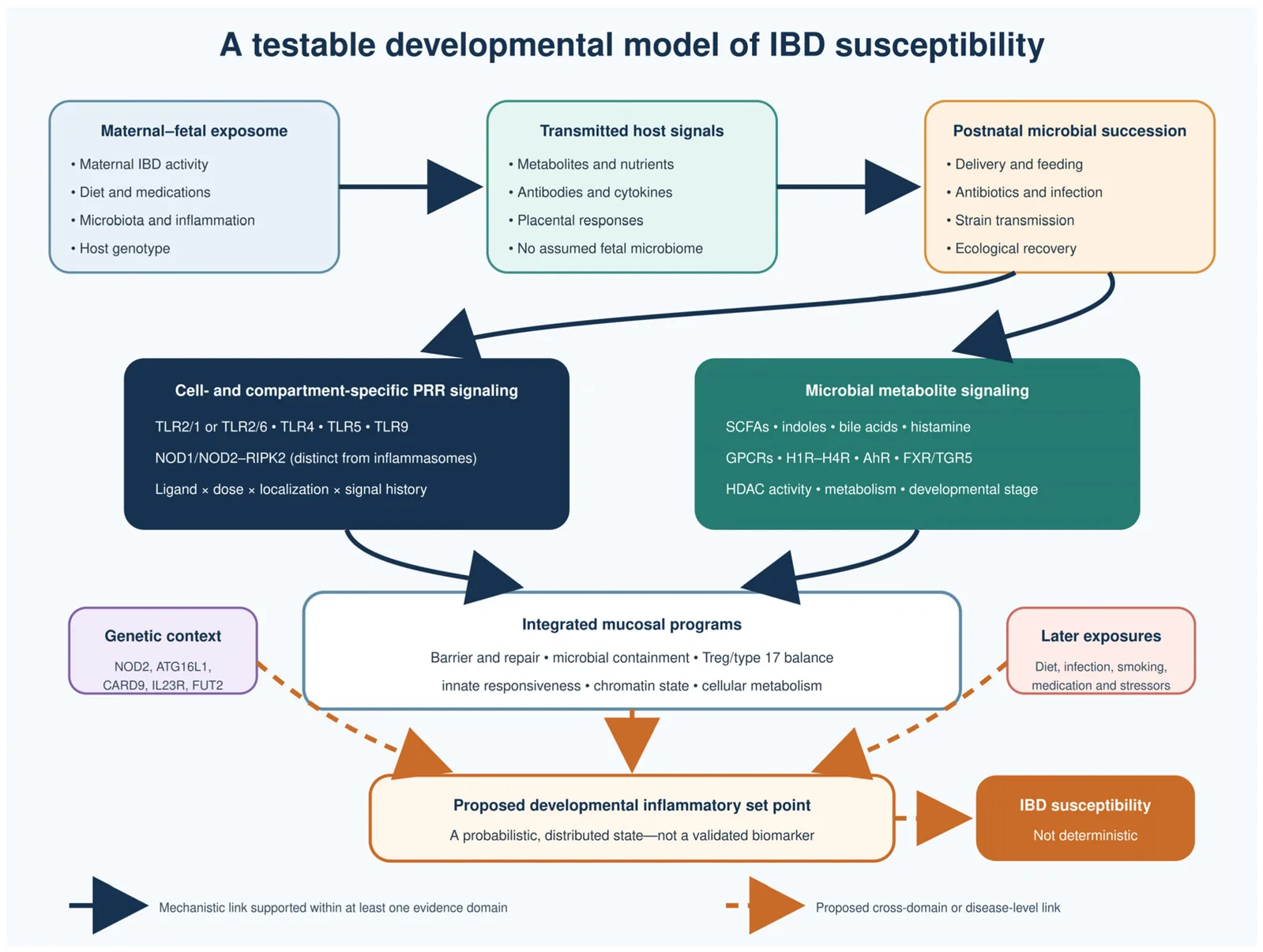
Testable developmental model linking the maternal–fetal exposome and postnatal microbial succession with cell- and compartment-specific PRR signaling, microbial metabolites including histamine, and integrated mucosal programs. Solid arrows indicate a mechanistic link supported within at least one evidence domain; dashed arrows indicate a proposed cross-domain or disease-level connection that requires validation. The developmental inflammatory set point is a heuristic, distributed state and not a validated biomarker. Abbreviations: AhR, aryl hydrocarbon receptor; FXR, farnesoid X receptor; GPCR, G-protein-coupled receptor; H1R–H4R, histamine receptors 1–4; HDAC, histone deacetylase; IBD, inflammatory bowel disease; PRR, pattern-recognition receptor; SCFA, short-chain fatty acid; TGR5, Takeda G-protein-coupled receptor 5; TLR, Toll-like receptor.

**Table 1:** Early-life factors and mechanisms potentially linking development with IBD susceptibility.

Early-Life Factor	Main Developmental Effects	Potential Link to IBD Susceptibility
Maternal–fetal environment	Maternal health, diet, microbiota, inflammation, and antibiotic exposure may influence fetal immune development and initial microbial colonization	Altered immune maturation and early host–microbiota interactions may modify later inflammatory responses
Mode of delivery and feeding	Delivery mode and breastfeeding/formula feeding influence initial microbial colonization and microbiome maturation	Changes in early microbial succession may affect immune tolerance and intestinal homeostasis
Antibiotics and early-life infections	Perturb microbial succession and provide inflammatory stimuli during immune maturation	Dysbiosis and altered immune calibration may influence subsequent susceptibility to intestinal inflammation
Microbiome maturation	Microbial succession and increasing community complexity shape host–microbiota interactions	Altered diversity, stability, or microbial functions may affect barrier integrity and immune regulation
Microbial metabolites	Short-Chain Fatty Acids, bile acids, and other metabolites provide metabolic signals during intestinal development	Changes in microbial metabolites may influence epithelial function, immune tolerance, and inflammatory responses
Epithelial and immune maturation	Development of the intestinal barrier, innate sensing, and adaptive immune regulation establishes mucosal homeostasis	Dysregulated maturation may alter the threshold for subsequent inflammatory responses
Epigenetic and immunometabolic programming	Early environmental signals may induce persistent changes in gene regulation and cellular metabolism	Such changes could modify long-term epithelial and immune responsiveness
Host genetic susceptibility	Genetic background influences responses to microbial and environmental signals	Developmental exposures may have different effects depending on genetic susceptibility, contributing to inter-individual variation
Developmental inflammatory set point	Integrated outcome of microbial, epithelial, metabolic, immune, and genetic signals during early life	Proposed framework in which early-life exposures may calibrate the magnitude and threshold of later intestinal inflammatory responses

**Table 2:** Testable predictions and falsification criteria for the developmental inflammatory set-point model.

Prediction	Candidate Operational Readout	Expected Observation	Observation That Would Falsify/Weaken the Prediction
Early-life exposure changes PRR responsiveness	Ex vivo LPS/flagellin challenge in age-matched cells; cytokines, antimicrobial/barrier responses	Reproducible cell- and ligand-specific response shift	No functional difference after age/current inflammation adjustment; receptor expression changes without functional change
Developmental conditioning leaves molecular memory	Longitudinal epithelial chromatin accessibility/transcriptomics	Persistent cell-specific molecular changes linked to functional response	No reproducible developmental signature or no relation to function
Set point modifies resilience	Serial FC + microbiome/metabolome + clinical trajectory	Altered recovery kinetics after later perturbation	No altered recovery; only transient nonspecific inflammation
Host genotype modifies developmental response	Pre-specified NOD2 × exposure analysis + functional intermediate phenotype	Replicable interaction with coherent mechanistic phenotype	Interaction absent on replication or no intermediate phenotype

**Table 3:** Evidence matrix for the proposed developmental pathway. Evidence categories are descriptive rather than hierarchical: direct human longitudinal evidence measures an exposure before later IBD; human mechanistic evidence measures an intermediate without later disease ascertainment; experimental causal evidence uses controlled perturbation; and experimental framework denotes a mechanism not yet connected to early-life human IBD. Abbreviations: AhR, aryl hydrocarbon receptor; H2R, histamine H2 receptor; IBD, inflammatory bowel disease; IEC, intestinal epithelial cell; IRAK1, interleukin-1 receptor-associated kinase 1; LPS, lipopolysaccharide; PBMC, peripheral blood mononuclear cell; PRR, pattern-recognition receptor; SCFA, short-chain fatty acid; TLR4, Toll-like receptor 4; Treg, regulatory T cell.

Component	Representative Observation	Evidence Type	Interpretation for Early Life IBD	Principal Limitation
Repeated antibiotic exposure	Population cohorts and meta-analysis associate repeated early antibiotic courses with later IBD [4,18,19].	Direct human longitudinal	Supports temporal association for an exposure that can disrupt microbial succession.	Confounding by infection, indication, health-care use, and prodromal disease; limited molecular mediation.
Maternal IBD	Maternal and infant microbiota or immune differences; transferred microbial communities altered immunity in germ-free mice [22,23].	Human mechanistic + experimental	Links a high-risk maternal context with infant biology.	Genetics, disease activity, medication, delivery, and feeding are difficult to separate; later offspring IBD not established.
Microbiota maturation	Longitudinal child cohorts show age-related trajectories and early effects of delivery and diet [17].	Human longitudinal microbiome	Supports a developmental trajectory and resilience framework.	Mostly healthy cohorts; composition does not establish immune programming or IBD causality.
Maternal microbial signals	Gestational colonization in mice changed offspring intestinal innate immune development [9].	Experimental causal	Shows prenatal microbial influence without requiring fetal colonization.	Human dose, transport, timing, and persistence remain uncertain.
Neonatal epithelial TLR4 tolerance	Postnatal LPS exposure induced IEC hyporesponsiveness and IRAK1 downregulation in mice [7].	Experimental causal	Demonstrates developmentally acquired PRR calibration.	Species-, cell-, ligand-, and compartment-specific; no direct link to later human IBD.
NOD2–RIPK2 signaling	IBD genetics and experimental work connect NOD2 with microbial sensing, autophagy, stem-cell protection, and repair [33,38,47].	Human genetics + experimental	Provides a plausible genotype-sensitive interface.	Developmental timing and exposure-specific effects in humans are not defined.
SCFA signaling	Butyrate and other commensal metabolites promote Treg differentiation and regulate barrier or immune functions [12,39,40].	Experimental causal + human association	Connects microbial function with chromatin, metabolism, and tolerance.	Concentration and cell context differ; fecal levels are poor proxies for tissue exposure; future IBD mediation unproven.
Indole and bile-acid metabolites	Defined metabolites regulate AhR–IL-22 or Treg/type 17 pathways in experimental systems [41,42,43].	Experimental causal	Identifies molecular routes from microbial metabolism to mucosal immunity.	Neonatal reference ranges, physiological tissue exposure, and longitudinal human outcomes are limited.
Histamine–H2R modulation of TLR signaling	Histamine–H2R signaling suppressed TLR2/4/5/9-induced cytokine responses in healthy PBMCs but was impaired in IBD; H2R loss or antagonism aggravated experimental colitis [44,45].	Human mechanistic + experimental	Demonstrates receptor-specific control of bacterial PRR outputs by a biogenic amine.	Post-diagnosis adult samples and experimental colitis; no evidence of prenatal or early-life programming.
Persistent innate or epithelial memory	Metabolic and chromatin remodeling can produce trained, tolerant, or tissue memory states [5,8,46].	Experimental framework	Explains how a transient exposure could have prolonged effects.	Lineage, duration, reversibility, and relevance to early-life human IBD require direct demonstration.

## Data Availability

No new data were created or analyzed in this study. Data sharing is not applicable to this article.

## Abbreviations

The following abbreviations are used in this manuscript:

IBD	Inflammatory Bowel Disease
PRR	Pattern-Recognition Receptor
H2R	Histamine H2 Receptor
CD	Crohn’s Disease
UC	Ulcerative Colitis
